# Diaqua­bis­(*N*,*N*-diethyl­pyridine-3-carboxamide-κ*N* ^1^)bis­{4-[2-(2,4-dioxopentan-3-yl­idene)hydrazin-1-yl]benzoato-κ*O*}copper(II)

**DOI:** 10.1107/S1600536811056200

**Published:** 2012-01-11

**Authors:** Rafiqa A. Alieva, Vusala I. Mardanova, Famil M. Chyraqov, Atash V. Gurbanov, Seik Weng Ng

**Affiliations:** a Department of Organic Chemistry, Baku State University, Baku, Azerbaijan; b Department of Chemistry, University of Malaya, 50603 Kuala Lumpur, Malaysia; c Chemistry Department, Faculty of Science, King Abdulaziz University, PO Box 80203 Jeddah, Saudi Arabia

## Abstract

In the title compound, [Cu(C_12_H_11_N_2_O_4_)_2_(C_10_H_14_N_2_O)_2_(H_2_O)_2_], the Cu^II^ atom lies on a center of inversion and is coordinated by carboxyl­ate O atoms, pyridine N atoms and two water mol­ecules in an elongated octa­hedral geometry. The pyridine ring is oriented at a dihedral angle of 74.83 (12)° with respect to the benzene ring. Intra­molecular O—H⋯O and N—H⋯O hydrogen bonding is observed. The water mol­ecule is a hydrogen-bond donor to the carbonyl O atom of an adjacent carboxyl­ate group, generating a chain running along the *a* axis. One of the ethyl groups is disordered over two sets of sites in a 0.787 (5):0.213 ratio.

## Related literature

For a related structure, see: Maharramov *et al.* (2011 ▶).
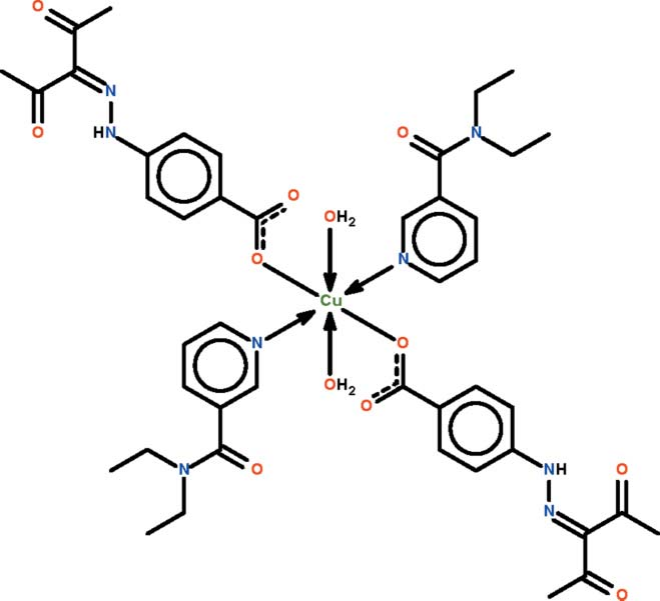


## Experimental

### 

#### Crystal data

[Cu(C_12_H_11_N_2_O_4_)_2_(C_10_H_14_N_2_O)_2_(H_2_O)_2_]
*M* *_r_* = 950.49
Triclinic, 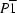

*a* = 7.7429 (4) Å
*b* = 8.7029 (4) Å
*c* = 19.0069 (9) Å
α = 85.695 (1)°
β = 83.218 (1)°
γ = 64.882 (1)°
*V* = 1151.12 (10) Å^3^
*Z* = 1
Mo *K*α radiation
μ = 0.54 mm^−1^
*T* = 296 K
0.30 × 0.30 × 0.20 mm

#### Data collection

Bruker SMART APEX diffractometer
Absorption correction: multi-scan (*SADABS*; Sheldrick, 1996 ▶) *T*_min_ = 0.854, *T*_max_ = 0.899
13589 measured reflections
5775 independent reflections
4780 reflections with *I* > 2σ(*I*)
*R*_int_ = 0.021

#### Refinement

*R*[*F* ^2^ > 2σ(*F* ^2^)] = 0.046
*wR*(*F* ^2^) = 0.134
*S* = 1.04
5775 reflections
305 parameters
38 restraints
H-atom parameters constrained
Δρ_max_ = 0.87 e Å^−3^
Δρ_min_ = −0.60 e Å^−3^

### 

Data collection: *APEX2* (Bruker, 2005 ▶); cell refinement: *SAINT* (Bruker, 2005 ▶); data reduction: *SAINT*; program(s) used to solve structure: *SHELXS97* (Sheldrick, 2008 ▶); program(s) used to refine structure: *SHELXL97* (Sheldrick, 2008 ▶); molecular graphics: *X-SEED* (Barbour, 2001 ▶); software used to prepare material for publication: *publCIF* (Westrip, 2010 ▶).

## Supplementary Material

Crystal structure: contains datablock(s) global, I. DOI: 10.1107/S1600536811056200/xu5431sup1.cif

Structure factors: contains datablock(s) I. DOI: 10.1107/S1600536811056200/xu5431Isup2.hkl

Additional supplementary materials:  crystallographic information; 3D view; checkCIF report

## Figures and Tables

**Table 1:** Selected bond lengths (Å)

Cu1—O1	1.9661 (15)
Cu1—N3	2.0151 (17)
Cu1—O1*W*	2.531 (2)

**Table 2:** Hydrogen-bond geometry (Å, °)

*D*—H⋯*A*	*D*—H	H⋯*A*	*D*⋯*A*	*D*—H⋯*A*
N1—H1⋯O3	0.88	1.88	2.558 (3)	132
O1w—H11⋯O2	0.84	2.06	2.712 (3)	135
O1w—H12⋯O5^i^	0.84	2.12	2.955 (3)	172

Symmetry code: (i) 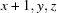.

**Fig. 1.**
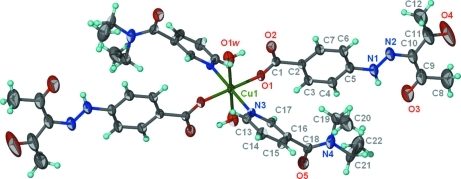
Thermal ellipsoid plot (Barbour, 2001) of Cu(H2O)2(C10H14N2O)2(C12H11N2O4)2 at the 50% probability level; hydrogen atoms are drawn as spheres of arbitrary radius. The disorder is not shown.

## supplementary crystallographic information

### Comment

We have been investigating the adducts of 3-diethylpridine-3-carboxamide with copper(II) salts; a previous study reported the copper bis(thienoylacetonate) adduct (Maharramov *et al.*, 2011). The reaction of the *N*-heterocycle with copper bis(4-[(2,4-dioxopentan-3-yl)diazenyl]benzoate yielded the title diaqua bis-adduct (Scheme I). The Cu^II^ atom in Cu(H_2_O)_2_(C_10_H_14_N_2_O)_2_(C_12_H_11_N_2_O_4_)_2_ lies on a center-of-inversion, and is coordinated to the O atom of the carboxylate ion, the N atom of the *N*-heterocycle and a water molecule in an all-*trans* octahedral geometry (Fig.1). The water molecule is hydrogen-bond donor to the the double-bond carbonyl O atom of adjacent carboxylate and *N*-heterocycle to generate a linear chain running along the *a*-axis of the triclinic unit cell (Table 1).

### Experimental

4-[(2,4-Dioxopentan-3-yl)diazenyl]benzoic acid and *N*,*N*-diethylpyridine-3-carboxamide were purchased from a chemical supplier. The carboxylic acid (0.0248 g, 0.01 mol) in water (50 ml) and an excess of cardioamine (5 ml) were added to a solution of copper acetate hydrate (0.0156 g, 0.01 mol) in water (50 ml). The green solution was filtered and then set aside for the growth of crystals within a week; yield 70%.

### Refinement

Carbon-bound H-atoms were placed in calculated positions [C–H 0.93 to 0.98 Å; *U*(H) 1.2 to 1.5*U*(C)] and were included in the refinement in the riding model approximation.

The water and amino- H-atoms were similarly positioned [O–H 0.84 and N–H 0.88 Å; *U*(H) 1.2 to 1.5*U*(N,*O*)].

Omitted were (0 0 1) and (1 1 0).

One of the ethyl chains of the neutral *N*-heterocycle is disordered over two positions in a 0.787 (5): 0.213 ratio. The pair of N–C distances were restrained to 0.01 Å of each other, and were the pair of C–C distances. The acetylacetate part of the substituted benzoate show somewhat elongated ellipsoids; the anisotropic temperature factors of these five atoms were restrained to be nearly isotropic.

### Crystal data

**Table:**

[Cu(C_12_H_11_N_2_O_4_)_2_(C_10_H_14_N_2_O)_2_(H_2_O)_2_]	*Z* = 1
*M**_r_* = 950.49	*F*(000) = 499
Triclinic, *P*1	*D*_x_ = 1.371 Mg m^−^^3^
Hall symbol: -P 1	Mo *K*α radiation, λ = 0.71073 Å
*a* = 7.7429 (4) Å	Cell parameters from 4939 reflections
*b* = 8.7029 (4) Å	θ = 2.6–28.4°
*c* = 19.0069 (9) Å	µ = 0.54 mm^−^^1^
α = 85.695 (1)°	*T* = 296 K
β = 83.218 (1)°	Prism, blue
γ = 64.882 (1)°	0.30 × 0.30 × 0.20 mm
*V* = 1151.12 (10) Å^3^	

### Data collection

**Table:**

Bruker SMART APEX diffractometer	5775 independent reflections
Radiation source: fine-focus sealed tube	4780 reflections with *I* > 2σ(*I*)
graphite	*R*_int_ = 0.021
φ and ω scans	θ_max_ = 28.4°, θ_min_ = 2.2°
Absorption correction: multi-scan (*SADABS*; Sheldrick, 1996)	*h* = −10→10
*T*_min_ = 0.854, *T*_max_ = 0.899	*k* = −11→11
13589 measured reflections	*l* = −25→25

### Refinement

**Table:**

Refinement on *F*^2^	Primary atom site location: structure-invariant direct methods
Least-squares matrix: full	Secondary atom site location: difference Fourier map
*R*[*F*^2^ > 2σ(*F*^2^)] = 0.046	Hydrogen site location: inferred from neighbouring sites
*wR*(*F*^2^) = 0.134	H-atom parameters constrained
*S* = 1.03	*w* = 1/[σ^2^(*F*_o_^2^) + (0.0788*P*)^2^ + 0.3231*P*] where *P* = (*F*_o_^2^ + 2*F*_c_^2^)/3
5775 reflections	(Δ/σ)_max_ = 0.001
305 parameters	Δρ_max_ = 0.87 e Å^−^^3^
38 restraints	Δρ_min_ = −0.60 e Å^−^^3^

### Fractional atomic coordinates and isotropic or equivalent isotropic displacement parameters (Å^2^)

**Table:**

	*x*	*y*	*z*	*U*_iso_*/*U*_eq_	Occ. (<1)
Cu1	0.5000	0.5000	0.5000	0.03554 (14)	
O1	0.3598 (2)	0.6157 (2)	0.41786 (8)	0.0404 (4)	
O2	0.5907 (3)	0.6225 (3)	0.33710 (10)	0.0507 (4)	
O3	−0.3219 (4)	0.6427 (5)	0.09450 (14)	0.1056 (11)	
O4	−0.1745 (8)	0.8431 (10)	−0.0897 (2)	0.227 (3)	
O5	0.1024 (3)	0.1348 (3)	0.40938 (10)	0.0572 (5)	
O1W	0.8200 (3)	0.4833 (3)	0.44344 (11)	0.0595 (5)	
H11	0.8057	0.5337	0.4036	0.089*	
H12	0.8971	0.3811	0.4384	0.089*	
N1	−0.0399 (3)	0.6766 (3)	0.13788 (11)	0.0530 (6)	
H1	−0.1407	0.6544	0.1492	0.064*	
N2	−0.0036 (4)	0.7205 (4)	0.07303 (12)	0.0596 (6)	
N3	0.5343 (3)	0.2781 (2)	0.46170 (9)	0.0332 (4)	
N4	0.2425 (3)	0.1170 (3)	0.29832 (12)	0.0548 (6)	
C1	0.4304 (3)	0.6252 (3)	0.35487 (12)	0.0349 (4)	
C2	0.3041 (3)	0.6390 (3)	0.29785 (11)	0.0344 (4)	
C3	0.1242 (3)	0.6404 (3)	0.31392 (11)	0.0374 (5)	
H3	0.0787	0.6335	0.3610	0.045*	
C4	0.0114 (3)	0.6519 (3)	0.26069 (12)	0.0415 (5)	
H4	−0.1087	0.6515	0.2718	0.050*	
C5	0.0797 (4)	0.6640 (3)	0.19063 (12)	0.0430 (5)	
C6	0.2596 (4)	0.6615 (4)	0.17352 (13)	0.0491 (6)	
H6	0.3050	0.6687	0.1264	0.059*	
C7	0.3706 (4)	0.6480 (4)	0.22731 (12)	0.0441 (5)	
H7	0.4922	0.6450	0.2161	0.053*	
C8	−0.4004 (7)	0.7049 (9)	−0.0225 (3)	0.129 (2)	
H8A	−0.4899	0.6572	−0.0064	0.193*	
H8B	−0.3189	0.6437	−0.0625	0.193*	
H8C	−0.4689	0.8220	−0.0361	0.193*	
C9	−0.2808 (5)	0.6920 (6)	0.03616 (17)	0.0761 (10)	
C10	−0.1170 (4)	0.7337 (5)	0.02394 (15)	0.0648 (8)	
C11	−0.0650 (7)	0.7972 (8)	−0.0451 (2)	0.1111 (18)	
C12	0.1209 (7)	0.8104 (8)	−0.0604 (2)	0.1074 (16)	
H12A	0.1154	0.8841	−0.1012	0.161*	
H12B	0.2216	0.6998	−0.0697	0.161*	
H12C	0.1460	0.8560	−0.0203	0.161*	
C13	0.6999 (3)	0.1395 (3)	0.46307 (12)	0.0377 (5)	
H13	0.7986	0.1436	0.4853	0.045*	
C14	0.7286 (4)	−0.0088 (3)	0.43248 (14)	0.0449 (5)	
H14	0.8446	−0.1038	0.4347	0.054*	
C15	0.5843 (4)	−0.0161 (3)	0.39847 (13)	0.0432 (5)	
H15	0.6025	−0.1147	0.3767	0.052*	
C16	0.4116 (3)	0.1270 (3)	0.39742 (11)	0.0345 (4)	
C17	0.3914 (3)	0.2698 (3)	0.43054 (11)	0.0342 (4)	
H17	0.2743	0.3644	0.4314	0.041*	
C18	0.2401 (3)	0.1260 (3)	0.36835 (12)	0.0372 (5)	
C19	0.3737 (6)	0.1654 (6)	0.24655 (19)	0.0557 (10)	0.787 (5)
H19A	0.4367	0.2170	0.2716	0.067*	0.787 (5)
H19B	0.2989	0.2488	0.2126	0.067*	0.787 (5)
C19'	0.4245 (16)	0.0383 (16)	0.2512 (7)	0.056*	0.213 (5)
H19C	0.4122	−0.0242	0.2134	0.067*	0.213 (5)
H19D	0.5315	−0.0346	0.2774	0.067*	0.213 (5)
C20	0.5230 (6)	0.0140 (7)	0.2076 (2)	0.0766 (14)	0.787 (5)
H20A	0.6053	0.0494	0.1754	0.115*	0.787 (5)
H20B	0.4611	−0.0353	0.1816	0.115*	0.787 (5)
H20C	0.5977	−0.0686	0.2411	0.115*	0.787 (5)
C20'	0.439 (3)	0.200 (2)	0.2245 (11)	0.077*	0.213 (5)
H20D	0.5386	0.2116	0.2463	0.115*	0.213 (5)
H20E	0.3193	0.2951	0.2361	0.115*	0.213 (5)
H20F	0.4691	0.1967	0.1740	0.115*	0.213 (5)
C21	0.0848 (4)	0.0938 (4)	0.27090 (17)	0.0589 (7)	
H21A	0.0448	0.0222	0.3041	0.071*	
H21B	0.1313	0.0360	0.2263	0.071*	
C22	−0.0839 (5)	0.2577 (5)	0.2597 (2)	0.0864 (12)	
H22A	−0.1821	0.2360	0.2419	0.130*	
H22B	−0.0458	0.3283	0.2261	0.130*	
H22C	−0.1323	0.3145	0.3039	0.130*	

### Atomic displacement parameters (Å^2^)

**Table:**

	*U*^11^	*U*^22^	*U*^33^	*U*^12^	*U*^13^	*U*^23^
Cu1	0.0516 (3)	0.0368 (2)	0.0268 (2)	−0.02437 (18)	−0.01424 (16)	0.00124 (14)
O1	0.0516 (9)	0.0441 (9)	0.0290 (8)	−0.0215 (8)	−0.0134 (7)	0.0016 (6)
O2	0.0466 (10)	0.0675 (12)	0.0454 (10)	−0.0294 (9)	−0.0143 (8)	0.0039 (8)
O3	0.0876 (18)	0.210 (4)	0.0570 (15)	−0.099 (2)	−0.0132 (13)	0.0126 (18)
O4	0.184 (4)	0.498 (9)	0.082 (2)	−0.224 (5)	−0.083 (3)	0.115 (4)
O5	0.0441 (10)	0.0895 (15)	0.0458 (10)	−0.0355 (10)	−0.0040 (8)	−0.0016 (10)
O1W	0.0570 (12)	0.0683 (13)	0.0441 (10)	−0.0167 (10)	−0.0075 (9)	−0.0026 (9)
N1	0.0507 (12)	0.0858 (17)	0.0326 (10)	−0.0375 (12)	−0.0123 (9)	0.0053 (10)
N2	0.0576 (14)	0.0968 (19)	0.0350 (11)	−0.0416 (14)	−0.0148 (10)	0.0078 (11)
N3	0.0383 (9)	0.0379 (9)	0.0295 (9)	−0.0203 (8)	−0.0083 (7)	−0.0015 (7)
N4	0.0488 (12)	0.0846 (17)	0.0400 (11)	−0.0339 (12)	−0.0082 (9)	−0.0130 (11)
C1	0.0445 (12)	0.0303 (10)	0.0326 (10)	−0.0164 (9)	−0.0117 (9)	0.0010 (8)
C2	0.0416 (11)	0.0357 (10)	0.0283 (10)	−0.0175 (9)	−0.0088 (9)	0.0011 (8)
C3	0.0415 (12)	0.0445 (12)	0.0265 (10)	−0.0183 (10)	−0.0045 (9)	0.0014 (8)
C4	0.0389 (12)	0.0551 (14)	0.0337 (11)	−0.0228 (11)	−0.0056 (9)	0.0023 (10)
C5	0.0456 (13)	0.0589 (14)	0.0300 (11)	−0.0259 (11)	−0.0113 (9)	0.0023 (10)
C6	0.0511 (14)	0.0775 (18)	0.0263 (11)	−0.0347 (14)	−0.0050 (10)	0.0025 (11)
C7	0.0432 (13)	0.0639 (15)	0.0329 (11)	−0.0303 (12)	−0.0062 (10)	0.0045 (10)
C8	0.106 (3)	0.240 (6)	0.085 (3)	−0.110 (4)	−0.048 (3)	0.023 (4)
C9	0.0617 (19)	0.131 (3)	0.0475 (17)	−0.050 (2)	−0.0140 (14)	−0.0019 (18)
C10	0.0590 (17)	0.110 (3)	0.0374 (14)	−0.0452 (18)	−0.0163 (12)	0.0070 (15)
C11	0.101 (3)	0.215 (6)	0.051 (2)	−0.098 (4)	−0.033 (2)	0.035 (3)
C12	0.105 (3)	0.186 (5)	0.061 (2)	−0.092 (3)	−0.014 (2)	0.023 (3)
C13	0.0352 (11)	0.0465 (12)	0.0352 (11)	−0.0197 (10)	−0.0072 (9)	−0.0019 (9)
C14	0.0363 (12)	0.0443 (12)	0.0493 (14)	−0.0105 (10)	−0.0062 (10)	−0.0089 (10)
C15	0.0452 (13)	0.0408 (12)	0.0454 (13)	−0.0180 (10)	−0.0036 (10)	−0.0129 (10)
C16	0.0381 (11)	0.0408 (11)	0.0306 (10)	−0.0216 (9)	−0.0051 (8)	−0.0030 (8)
C17	0.0357 (11)	0.0363 (10)	0.0336 (10)	−0.0169 (9)	−0.0080 (9)	−0.0007 (8)
C18	0.0394 (12)	0.0387 (11)	0.0377 (11)	−0.0189 (9)	−0.0076 (9)	−0.0045 (9)
C19	0.063 (2)	0.076 (3)	0.0377 (17)	−0.038 (2)	−0.0095 (16)	0.0061 (16)
C20	0.066 (3)	0.101 (4)	0.051 (2)	−0.026 (2)	0.004 (2)	−0.001 (2)
C21	0.0562 (16)	0.0630 (17)	0.0628 (18)	−0.0236 (14)	−0.0216 (14)	−0.0170 (14)
C22	0.078 (2)	0.076 (2)	0.100 (3)	−0.0175 (19)	−0.039 (2)	−0.017 (2)

### Geometric parameters (Å, °)

**Table:**

Cu1—O1	1.9661 (15)	C8—H8B	0.9600
Cu1—O1^i^	1.9661 (15)	C8—H8C	0.9600
Cu1—N3	2.0151 (17)	C9—C10	1.450 (5)
Cu1—N3^i^	2.0151 (17)	C10—C11	1.466 (5)
Cu1—O1W	2.531 (2)	C11—C12	1.485 (6)
O1—C1	1.268 (3)	C12—H12A	0.9600
O2—C1	1.239 (3)	C12—H12B	0.9600
O3—C9	1.215 (4)	C12—H12C	0.9600
O4—C11	1.195 (5)	C13—C14	1.376 (3)
O5—C18	1.222 (3)	C13—H13	0.9300
O1W—H11	0.8400	C14—C15	1.380 (3)
O1W—H12	0.8400	C14—H14	0.9300
N1—N2	1.297 (3)	C15—C16	1.389 (3)
N1—C5	1.411 (3)	C15—H15	0.9300
N1—H1	0.8800	C16—C17	1.377 (3)
N2—C10	1.322 (3)	C16—C18	1.500 (3)
N3—C13	1.338 (3)	C17—H17	0.9300
N3—C17	1.345 (3)	C19—C20	1.507 (6)
N4—C18	1.337 (3)	C19—H19A	0.9700
N4—C21	1.476 (3)	C19—H19B	0.9700
N4—C19'	1.493 (10)	C19'—C20'	1.506 (11)
N4—C19	1.497 (4)	C19'—H19C	0.9700
C1—C2	1.509 (3)	C19'—H19D	0.9700
C2—C3	1.386 (3)	C20—H20A	0.9600
C2—C7	1.387 (3)	C20—H20B	0.9600
C3—C4	1.384 (3)	C20—H20C	0.9600
C3—H3	0.9300	C20'—H20D	0.9600
C4—C5	1.387 (3)	C20'—H20E	0.9600
C4—H4	0.9300	C20'—H20F	0.9600
C5—C6	1.384 (4)	C21—C22	1.491 (4)
C6—C7	1.379 (3)	C21—H21A	0.9700
C6—H6	0.9300	C21—H21B	0.9700
C7—H7	0.9300	C22—H22A	0.9600
C8—C9	1.500 (5)	C22—H22B	0.9600
C8—H8A	0.9600	C22—H22C	0.9600
			
O1—Cu1—O1^i^	180.00 (5)	C10—C11—C12	121.0 (3)
O1—Cu1—N3	88.22 (7)	C11—C12—H12A	109.5
O1^i^—Cu1—N3	91.78 (7)	C11—C12—H12B	109.5
O1—Cu1—N3^i^	91.78 (7)	H12A—C12—H12B	109.5
O1^i^—Cu1—N3^i^	88.22 (7)	C11—C12—H12C	109.5
N3—Cu1—N3^i^	180.00 (9)	H12A—C12—H12C	109.5
O1—Cu1—O1W	94.78 (7)	H12B—C12—H12C	109.5
O1^i^—Cu1—O1W	85.22 (7)	N3—C13—C14	121.9 (2)
N3—Cu1—O1W	94.61 (7)	N3—C13—H13	119.1
N3^i^—Cu1—O1W	85.39 (7)	C14—C13—H13	119.1
C1—O1—Cu1	127.21 (15)	C13—C14—C15	119.7 (2)
Cu1—O1W—H11	109.5	C13—C14—H14	120.2
Cu1—O1W—H12	109.5	C15—C14—H14	120.2
H11—O1W—H12	109.5	C14—C15—C16	118.6 (2)
N2—N1—C5	121.0 (2)	C14—C15—H15	120.7
N2—N1—H1	119.5	C16—C15—H15	120.7
C5—N1—H1	119.5	C17—C16—C15	118.5 (2)
N1—N2—C10	120.8 (3)	C17—C16—C18	118.7 (2)
C13—N3—C17	118.55 (18)	C15—C16—C18	122.47 (19)
C13—N3—Cu1	121.68 (14)	N3—C17—C16	122.6 (2)
C17—N3—Cu1	119.68 (15)	N3—C17—H17	118.7
C18—N4—C21	118.4 (2)	C16—C17—H17	118.7
C18—N4—C19'	122.4 (6)	O5—C18—N4	122.2 (2)
C21—N4—C19'	111.2 (6)	O5—C18—C16	119.0 (2)
C18—N4—C19	122.2 (2)	N4—C18—C16	118.8 (2)
C21—N4—C19	118.0 (2)	N4—C19—C20	111.8 (4)
O2—C1—O1	125.9 (2)	N4—C19—H19A	109.3
O2—C1—C2	118.8 (2)	C20—C19—H19A	109.3
O1—C1—C2	115.3 (2)	N4—C19—H19B	109.3
C3—C2—C7	118.9 (2)	C20—C19—H19B	109.3
C3—C2—C1	121.8 (2)	H19A—C19—H19B	107.9
C7—C2—C1	119.3 (2)	N4—C19'—C20'	97.5 (11)
C4—C3—C2	120.8 (2)	N4—C19'—H19C	112.3
C4—C3—H3	119.6	C20'—C19'—H19C	112.3
C2—C3—H3	119.6	N4—C19'—H19D	112.3
C3—C4—C5	119.2 (2)	C20'—C19'—H19D	112.3
C3—C4—H4	120.4	H19C—C19'—H19D	109.9
C5—C4—H4	120.4	C19—C20—H20A	109.5
C6—C5—C4	120.9 (2)	C19—C20—H20B	109.5
C6—C5—N1	121.6 (2)	H20A—C20—H20B	109.5
C4—C5—N1	117.5 (2)	C19—C20—H20C	109.5
C7—C6—C5	119.0 (2)	H20A—C20—H20C	109.5
C7—C6—H6	120.5	H20B—C20—H20C	109.5
C5—C6—H6	120.5	C19'—C20'—H20D	109.5
C6—C7—C2	121.2 (2)	C19'—C20'—H20E	109.5
C6—C7—H7	119.4	H20D—C20'—H20E	109.5
C2—C7—H7	119.4	C19'—C20'—H20F	109.5
C9—C8—H8A	109.5	H20D—C20'—H20F	109.5
C9—C8—H8B	109.5	H20E—C20'—H20F	109.5
H8A—C8—H8B	109.5	N4—C21—C22	112.7 (3)
C9—C8—H8C	109.5	N4—C21—H21A	109.1
H8A—C8—H8C	109.5	C22—C21—H21A	109.1
H8B—C8—H8C	109.5	N4—C21—H21B	109.1
O3—C9—C10	120.1 (3)	C22—C21—H21B	109.1
O3—C9—C8	118.5 (4)	H21A—C21—H21B	107.8
C10—C9—C8	121.3 (3)	C21—C22—H22A	109.5
N2—C10—C9	124.0 (3)	C21—C22—H22B	109.5
N2—C10—C11	114.1 (3)	H22A—C22—H22B	109.5
C9—C10—C11	121.9 (3)	C21—C22—H22C	109.5
O4—C11—C10	120.1 (4)	H22A—C22—H22C	109.5
O4—C11—C12	118.8 (4)	H22B—C22—H22C	109.5
			
N3—Cu1—O1—C1	−77.11 (18)	N2—C10—C11—O4	−166.7 (7)
N3^i^—Cu1—O1—C1	102.89 (18)	C9—C10—C11—O4	12.6 (10)
O1W—Cu1—O1—C1	17.37 (18)	N2—C10—C11—C12	11.7 (7)
C5—N1—N2—C10	−179.4 (3)	C9—C10—C11—C12	−169.1 (5)
O1—Cu1—N3—C13	138.76 (18)	C17—N3—C13—C14	1.2 (3)
O1^i^—Cu1—N3—C13	−41.24 (18)	Cu1—N3—C13—C14	−175.28 (18)
O1W—Cu1—N3—C13	44.11 (18)	N3—C13—C14—C15	0.8 (4)
O1—Cu1—N3—C17	−37.72 (17)	C13—C14—C15—C16	−1.3 (4)
O1^i^—Cu1—N3—C17	142.28 (17)	C14—C15—C16—C17	−0.2 (4)
O1W—Cu1—N3—C17	−132.37 (16)	C14—C15—C16—C18	−173.6 (2)
Cu1—O1—C1—O2	−28.8 (3)	C13—N3—C17—C16	−2.8 (3)
Cu1—O1—C1—C2	150.44 (15)	Cu1—N3—C17—C16	173.76 (16)
O2—C1—C2—C3	179.1 (2)	C15—C16—C17—N3	2.3 (3)
O1—C1—C2—C3	−0.2 (3)	C18—C16—C17—N3	176.0 (2)
O2—C1—C2—C7	0.1 (3)	C21—N4—C18—O5	−8.4 (4)
O1—C1—C2—C7	−179.2 (2)	C19'—N4—C18—O5	−154.6 (7)
C7—C2—C3—C4	−0.5 (4)	C19—N4—C18—O5	158.5 (3)
C1—C2—C3—C4	−179.5 (2)	C21—N4—C18—C16	172.2 (2)
C2—C3—C4—C5	−0.7 (4)	C19'—N4—C18—C16	26.0 (7)
C3—C4—C5—C6	1.2 (4)	C19—N4—C18—C16	−20.9 (4)
C3—C4—C5—N1	−179.6 (2)	C17—C16—C18—O5	−64.4 (3)
N2—N1—C5—C6	−14.1 (4)	C15—C16—C18—O5	109.1 (3)
N2—N1—C5—C4	166.8 (3)	C17—C16—C18—N4	115.1 (3)
C4—C5—C6—C7	−0.5 (4)	C15—C16—C18—N4	−71.5 (3)
N1—C5—C6—C7	−179.7 (3)	C18—N4—C19—C20	112.8 (4)
C5—C6—C7—C2	−0.7 (4)	C21—N4—C19—C20	−80.2 (4)
C3—C2—C7—C6	1.2 (4)	C19'—N4—C19—C20	9.6 (9)
C1—C2—C7—C6	−179.7 (2)	C18—N4—C19'—C20'	−99.5 (11)
N1—N2—C10—C9	−3.8 (6)	C21—N4—C19'—C20'	112.1 (10)
N1—N2—C10—C11	175.4 (4)	C19—N4—C19'—C20'	3.3 (9)
O3—C9—C10—N2	0.6 (7)	C18—N4—C21—C22	86.4 (4)
C8—C9—C10—N2	−178.2 (4)	C19'—N4—C21—C22	−123.8 (6)
O3—C9—C10—C11	−178.6 (5)	C19—N4—C21—C22	−81.0 (4)
C8—C9—C10—C11	2.7 (7)		

Symmetry codes: (i) −*x*+1, −*y*+1, −*z*+1.

### Hydrogen-bond geometry (Å, °)

**Table:**

*D*—H···*A*	*D*—H	H···*A*	*D*···*A*	*D*—H···*A*
N1—H1···O3	0.88	1.88	2.558 (3)	132
O1w—H11···O2	0.84	2.06	2.712 (3)	135
O1w—H12···O5^ii^	0.84	2.12	2.955 (3)	172

Symmetry codes: (ii) *x*+1, *y*, *z*.
